# Increasing risk of revision due to deep infection after hip arthroplasty: A study on 97,344 primary total hip replacements in the Norwegian Arthroplasty Register from 1987 to 2007

**DOI:** 10.3109/17453670903506658

**Published:** 2009-12-04

**Authors:** Håvard Dale, Geir Hallan, Birgitte Espehaug, Leif I Havelin, Lars B Engesæter

**Affiliations:** ^1^The Norwegian Arthroplasty Register, Department of Orthopaedic Surgery, Haukeland University Hospital; ^2^Institute of Surgical Sciences, University of Bergen, Bergen, Norway

## Abstract

**Background and purpose** Over the decades, improvements in surgery and perioperative routines have reduced the incidence of deep infections after total hip arthroplasty (THA). There is, however, some evidence to suggest that the incidence of infection is increasing again. We assessed the risk of revision due to deep infection for primary THAs reported to the Norwegian Arthroplasty Register (NAR) over the period 1987–2007.

**Method** We included all primary cemented and uncemented THAs reported to the NAR from September 15, 1987 to January 1, 2008 and performed adjusted Cox regression analyses with the first revision due to deep infection as endpoint. Changes in revision rate as a function of the year of operation were investigated.

**Results** Of the 97,344 primary THAs that met the inclusion criteria, 614 THAs had been revised due to deep infection (5-year survival 99.46%). Risk of revision due to deep infection increased throughout the period studied. Compared to the THAs implanted in 1987–1992, the risk of revision due to infection was 1.3 times higher (95%CI: 1.0–1.7) for those implanted in 1993–1997, 1.5 times (95% CI: 1.2–2.0) for those implanted in 1998–2002, and 3.0 times (95% CI: 2.2–4.0) for those implanted in 2003–2007. The most pronounced increase in risk of being revised due to deep infection was for the subgroup of uncemented THAs from 2003–2007, which had an increase of 5 times (95% CI: 2.6–11) compared to uncemented THAs from 1987–1992.

**Interpretation** The incidence of deep infection after THA increased during the period 1987–2007. Concomitant changes in confounding factors, however, complicate the interpretation of the results.

## Introduction

Improvements in surgical technique, perioperative routines, and prophylactic measures have reduced the incidence of infection from 5–10% in the late 1960s (Charnley 1972) to around 1% (Gaine et al. 2000, Zimmerli and Ochsner 2003, Phillips et al. 2006). There is, however, some evidence to suggest that the incidence of infection is increasing (Kurtz et al. 2008). Few publications have addressed time trends concerning postoperative infections after THAs, and large numbers of primary THAs are required to show changes in risk of infection. We assessed whether there have been any changes in risk of revision due to deep infection for THAs reported to the Norwegian Arthroplasty Register over the last 2 decades.

## Patients and methods

Since its inception on September 15, 1987, the Norwegian Arthroplasty Register (NAR) has registered detailed data on primary THAs and THA revisions in Norway. The data gathered include information on patient identity, date of operation, indication for surgery, type of implant, method of fixation, duration of surgery, type of operating room ventilation, and the type of antibiotic prophylaxis used. The unique identification number of each inhabitant of Norway is used to link the primary THA to any revision (Havelin et al. 2000). Revision due to deep infection of the implant is defined as removal or exchange of the whole or parts of the prosthesis, with deep infection reported as the diagnosis. Isolated soft tissue revisions are not reported to the register. The register form is filled in by the surgeon immediately after surgery.

The period of inclusion and observation in this study was from the start of the NAR on September 15, 1987 to January 1, 2008. For this time period, the NAR contained data on 110,882 primary THAs. In order to have homogeneous subgroups concerning type of fixation, 4,392 hybrids and 3,727 reversed hybrids were excluded. 3,730 arthroplasties had incomplete data on fixation method or were registered with different brands of cement for different components, and were also excluded. 1,689 additional THAs were excluded because of missing values for other adjustment variables. There were 97,344 THAs with complete information where both components were either cemented or uncemented, and these were eligible for analysis.

All THAs were followed until their first revision due to deep infection or revision for other causes, until date of death or emigration of the patient, or until January 1, 2008. Thus, follow-up was 0–20 years. 4 time periods were compared: 1987–1992, 1993–1997, 1998–2002, and 2003–2007, with subanalyses on cemented and uncemented THAs.

As a control, we performed a subanalysis on Charnley prostheses fixed with antibiotic-loaded bone cement and given antibiotic prophylaxis systemically. This prosthesis was the most used in Norway from 1987 to 2008, and it was used extensively throughout the whole period of observation.

### Statistics

Survival analyses were performed with a Cox regression model, with time period as main risk factor and revision due to deep infection as the endpoint. Revision rate ratios (RRs) for the time periods are presented with 95% confidence interval (CI) and p-values relative to the first time period. We adjusted for differences over time concerning sex, age (< 40, 40–59, 60–69, 70–79, ≥ 80 years), diagnosis (osteoarthritis, inflammatory disease, other), monoblock or modular prosthesis, type of fixation (uncemented, cemented with cement containing or not containing antibiotics), antibiotic prophylaxis systemically (yes, no), type of operation room ventilation (ordinary, laminar flow, greenhouse), and duration of surgery (<70, 70–99, 100–129, or ≥ 130 min). Cox regression analyses with time period as stratification factor were used to construct cumulative revision curves (1 minus cumulative survival) at mean values of the covariates, and to assess 5-year survival percentages. We also performed a separate Cox analysis with revision due to aseptic loosening as endpoint for all THAs, in order to be able to compare these findings with our findings for revision due to deep infection. Furthermore, to ensure similar potential follow-up for operations in all time periods, additional analyses were performed with follow-up restricted to 0–5 years.

We also investigated changes in the revision rate due to deep infection as a function of year of operation. These analyses gave a graphical display of the relationship based on a generalized additive model for survival data (Hastie and Tibshirani 1990). The curves are presented with 95% CI.

Risk ratio analyses were performed for the different risk factors and prophylactic measures for each time period separately, and for the whole 20-year period adjusted for year of primary surgery.

Values of p less than 0.05 were considered statistically significant. We used SPSS software version 15.0.

## Results

97,344 primary THAs in 79,820 patients met the inclusion criteria for this study. 614 first revisions due to deep infection were reported in 610 patients. The 5-year survival was 99.46% with revision due to deep infection as endpoint.

The distribution of patient characteristics such as sex, age, and diagnosis of patients undergoing primary THA was stable throughout the period studied (Table 1), except for the group of primary uncemented THAs, where mean age increased from 52 (SD 12) in 1987–1992 to 61 (SD 13) in 2003–2007. There was a shift from monoblock towards modular THAs (Table 1). Duration of surgery decreased slightly, whereas the use of an operating room with laminar air flow increased through the 4 time periods (Table 1). Antibiotic-loaded bone cement was used more extensively, and cement containing antibiotics was used in most cemented THAs towards the end of the study period (Table 1). Except during the first time period, prophylactic antibiotics were administered systemically in almost all operations (Table 1).

**Table 1.** Primary THAs included over the four 5-year time periods

Variable	1987–1992	1993–1997	1998–2002	2003–2007
No. of THAs	20,913	22,519	26,230	27,682
Sex (%)				
Male	30	30	29	31
Female	70	70	71	69
Age (%)				
< 40	2	2	2	1
40–59	15	14	15	14
60–69	31	27	26	26
70–79	41	43	41	39
≥ 80	12	14	17	19
Diagnosis (%)				
Osteoarthritis	67	70	73	77
Inflammatory	4	4	4	3
Other	29	26	23	20
Prosthesis (%)				
Monoblock	58	53	38	22
Modular	42	47	62	78
Duration (min) of surgery (%)				
< 70	11	10	11	15
70–99	41	45	45	45
100–129	31	31	31	29
≥ 130	18	15	13	12
Operation room ventilation (%)				
“Greenhouse”	12	2	1	1
Laminar flow	29	45	53	56
Ordinary	59	53	46	44
Antibiotic prophylaxis systemically (%)				
No	8	0	0	0
Yes	92	100	100	100
Method of fixation (%)				
Uncemented	15	15	14	16
Cement				
with antibiotics	38	56	82	83
without antibiotics	48	29	4	2

### Time trend: deep infection

**Figure 1.**
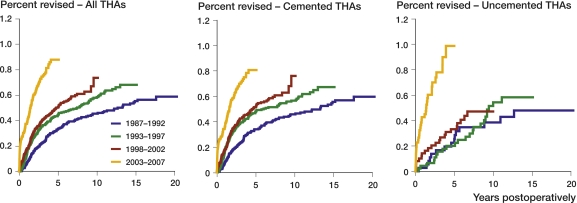
Percentage revision due to deep infection, for all THAs, for cemented THAs, and for uncemented THAs, for 4 periods of primary surgery, adjusted for sex, age, diagnosis, prosthesis, operation room ventilation, duration of operation, and antibiotic prophylaxis.

**Figure 2.**
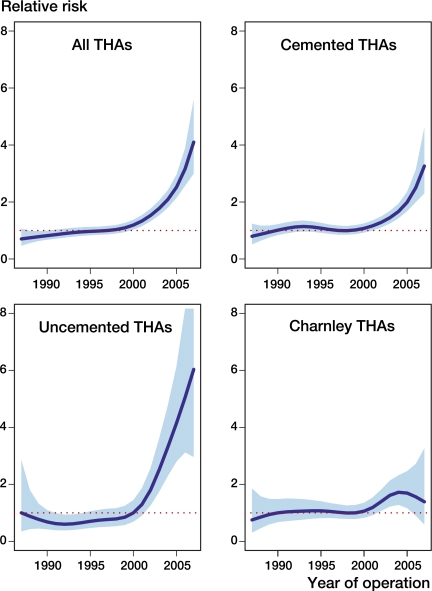
Graphical display of the relationship between year of primary surgery and risk of revision due to deep infection (with 95% CI) for all THAs, cemented THAs, uncemented THAs, and Charnley THAs with uniform antibiotic prophylaxis, adjusted for sex, age, diagnosis, prosthesis, operation room ventilation, duration of operation, type of fixation, and antibiotic prophylaxis.

For all primary THAs, we found an increase in the risk of revision due to deep infection, compared to the time period 1987–1992, for all 3 of the other consecutive time periods. The risk of revision due to infection was 1.3 times higher for 1993–1997, 1.5 times higher for 1998–2002, and 3.0 times higher for 2003–2007, respectively (Table 2, Figure 1). The risk of infection increased throughout the whole period of observation (Figure 2).

**Table 2.** Risk ratios and 5-year survival estimates for revision due to deep infection. The risk ratios and survival estimates are adjusted for sex, age, diagnosis, prosthesis, operation room ventilation, duration of operation, and antibiotic prophylaxis

Prosthesis	Time period	No. of THAs included	No. of THAs revised due infection	Risk ratio	p-value	95% CI	5-year survival
All THAs	1987–1992	20,913	134	1			99.7
	1993–1997	22,519	156	1.3	0.03	1.0–1.7	99.6
	1998–2002	26,230	150	1.5	0.003	1.2–2.0	99.5
	2003–2007	27,682	174	3.0	< 0.001	2.2–4.0	99.1
Cemented THAs	1987–1992	17,867	119	1			99.7
	1993–1997	19,191	133	1.3	0.04	1.0–1.7	99.5
	1998–2002	22,558	129	1.5	0.008	1.1–2.1	99.5
	2003–2007	23,380	136	2.7	< 0.001	1.9–3.7	99.2
Uncemented THAs	1987–1992	3,046	15	1			99.8
	1993–1997	3,328	23	1.2	0.6	0.6–2.4	99.8
	1998–2002	3,672	21	1.4	0.3	0.7–2.9	99.6
	2003–2007	4,302	38	5.3	< 0.001	2.6–10.7	98.9
Charnley with antibiotics in the cement and systemically	1987–1992	4,321	26	1			99.7
	1993–1997	7,776	46	1.1	0.6	0.7–1.9	99.6
	1998–2002	9,301	44	1.1	0.9	0.6–1.8	99.6
	2003–2007	5,925	37	2.0	0.02	1.1–3.5	99.3

In the cemented group of primary THAs, with revision due to deep infection as endpoint, we found the same pattern of gradual increase in revision risk over time (Table 2, Figures 1 and 2). This was also found in the subgroup of Charnley prostheses fixed with antibiotic-loaded bone cement and given antibiotic prophylaxis systemically (Table 2).

Uncemented THAs had a 5.3 times higher risk of being revised due to deep infection in the last time period compared to 1987–1992 (Table 2, Figure 1). The 5-year survival (98.94%) was also inferior to that of the cemented group (99.20%) for this period (difference = 0.26%, CI: 0.22–0.30, p < 0.001). The increase in risk of revision due to deep infection was most pronounced after the year 2000 for uncemented THAs (Figure 2).

We had 0–20 years of follow-up in our study, but maximum follow-up varied for THAs in the different time periods. To determine whether this would have influenced the results, analyses were performed including only 0–5 year follow-up for each group. This did not change the findings.

### Time trend: aseptic loosening

**Figure 3.**
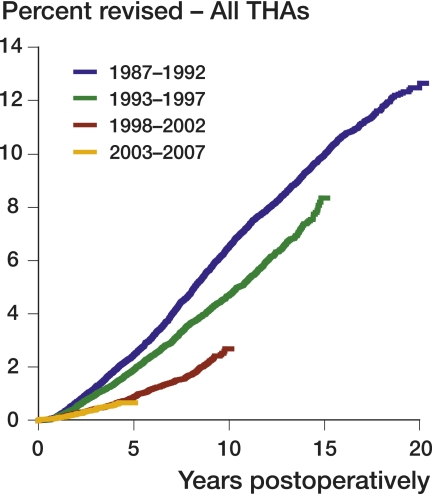
Percentage revision due to aseptic loosening, for all THAs, for 4 periods of primary surgery, adjusted for sex, age, diagnosis, prosthesis, operation room ventilation, duration of operation, type of fixation, and antibiotic prophylaxis.

There were 4,437 primary THAs revised due to aseptic loosening in the entire period studied. The percentage revised due to aseptic loosening decreased significantly throughout the period (Figure 3). Relative to the time period 1987–1992, the risk of revision due to aseptic loosening was 0.4 times (CI: 0.3–0.4) for the time period 1998–2002 (p < 0.001) and 0.3 times (CI: 0.3–0.4) for 2003–2007 (p < 0.001). There was no statistically significant difference between the 2 latter time periods concerning risk of revision due to aseptic loosening (Figure 3).

### Impact of risk factors and prophylactic measures on deep infection

We assessed the effect of the different risk factors and prophylactic measures that were adjusted for in the Cox analysis. These factors were adjusted for year of index surgery to adjust for unknown confounding and time-dependent factors.

Male sex was a significant risk factor for revision due to deep infection, but age and diagnosis did not influence the risk (Table 3). Laminar air flow was associated with a higher risk of revision due to infection postoperatively compared to ordinary ventilation (Table 3). There was also a higher risk of revision due to infection in the groups with an operating time of more than 100 min (Table 3). Uncemented THAs and THAs implanted with plain cement had a statistically significantly higher risk of revision due to infection compared to cemented THAs fixed with antibiotic-loaded cement (Table 3). Exclusion of monoblock prostheses from the cemented group did not alter these findings. In the small group of patients who did not receive antibiotic prophylaxis systemically, we found a 60% higher risk of THAs being revised due to infection (Table 3). Subanalyses of the risk factors and prophylactic measures performed for each time period separately showed similar effects in all 4 time periods.

**Table 3.** Number of primary THAs included and number of reported first revisions due to deep infection. Adjusted risk ratio estimates for sex, age, diagnosis, type of prosthesis, duration of operation, operation room ventilation, antibiotic prophylaxis systemically, and type of fixation. The risk factors are adjusted for all the other risk factors in addition to year of surgery

	No. of included	THAs revised due to infection	Risk ratio	p-value	95% CI
Sex					
Male	29,216	311	2.5	< 0.001	2.1–2.9
Female	68,128	303	1		
Age					
< 40	1,721	9	0.5	0.1	0.3–1.1
40–59	14,240	95	0.8	0.2	0.6–1.1
60–69	26,336	196	1.1	0.3	0.9–1.3
70–79	39,812	241	1		
≥ 80	15,235	73	0.9	0.5	0.7–1.2
Diagnosis					
Osteoarthritis	70,134	440	1		
Inflammatory	3,522	22	1.1	0.6	0.7–1.7
Other	23,688	152	1.2	0.1	1.0–1.4
Prosthesis					
Modular	57,374	332	0.8	0.1	0.7–1.0
Monoblock	39,970	282	1		
Duration of surgery, min					
< 70	11,334	55	0.9	0.5	0.7–1.2
70–99	42,700	236	1		
100–129	29,679	211	1.3	0.01	1.0–1.5
≥ 130	13,631	112	1.5	0.001	1.2–1-9
Operation room ventilation					
Greenhouse	3,386	30	1.3	0.2	0.9–2.0
Laminar flow	45,620	324	1.3	0.006	1.1–1.5
Ordinary	48,338	260	1		
Antibiotic prophylaxis systemically					
No	1,820	15	1.6	0.1	0.9–2.7
Yes	95,524	599	1		
Method of fixation					
Uncemented	14,348	97	1.4	0.03	1.0–1.8
Cement					
with antibiotics	65,005	360	1		
without antibiotics	17,991	157	1.9	< 0.001	1.5–2.3

Comparison of unadjusted and adjusted risk estimates for the 4 time periods showed that different covariates acted as confounders for cemented and uncemented THAs. Comparing the first and the last time period for cemented THAs, the risk of revision due to infection increased from 1.8 (CI: 1.4–2.3) (p < 0.001) to 2.7 (CI: 1.9–3.7) (p < 0.001). This change was mainly due to adjustment for use of cement containing antibiotics and explained by increased use over time and the protective ability of cement containing antibiotics. There was also a trend of shorter duration of surgery having a protective effect on cemented THAs. For Charnley prostheses inserted with cement containing antibiotics, the effect of adjustment was negligible. For uncemented THAs, the risk of revision due to infection was reduced from 5.7 (CI: 2.9–11.2) (p < 0.001) to 5.3 (CI: 2.6–10.7) (p < 0.001) for the last time period relative to the first. The decrease was caused by adjustment for sex.

## Discussion

Our main finding was an increased risk of revision due to deep infection after primary THA for the 3 consecutive 5-year periods after 1987–1992. The most pronounced increase was for the last time period. The increase was particularly high in the subgroup of uncemented THAs.

We have found no reports on an increased risk of infection for primary THAs. Kurtz et al. (2008) report a 2-fold increase in overall incidence of deep infection after THA from 0.66% in 1990 to 1.23% in 2004. This study on “total infection burden” was based on aggregated data, and both primary and revision arthroplasties were included in the analyses. For primary THAs only, they found a reduced incidence of infection. Mannien et al. (2008) also reported a 60% decrease in surgical site infection after THA between 1996 and 2006 in the Dutch national nosocomial surveillance network (PREZIES). The Cochrane collaboration has not evaluated THA infections.

To our knowledge, the finding that uncemented THAs have shown a larger increase in infection rate than cemented THAs in recent years has not been described previously. The most pronounced increase in risk of revision due to infection in uncemented THAs was after the year 2000. Engesaeter et al. (2006) concluded in their study from the Norwegian Arthroplasty Register, including THAs from the period 1987–2003, that the risk of revision due to infection was the same for uncemented THAs and THAs fixed with cement-containing antibiotics. THAs fixed with cement without antibiotics had a higher risk of deep infection. Based on our study, we have reason to believe that there is now a trend towards higher susceptibility to deep infection for uncemented THAs than for THAs implanted with cement-containing antibiotics. This confirms earlier findings that antibiotic-loaded bone cement protects against infection (Engesaeter et al. 2003, Block and Stubbs 2005, Parvizi et al. 2008b).

One possible explanation for the increased risk of infection could be that THA is now performed on patients with more comorbidity. Obesity and diabetes have an increasing incidence in the population, and these conditions are both risk factors for postoperative surgical site infections (Olsen et al. 2008, Pulido et al. 2008). These factors are not reported to our register, but if our material is similar to the general population, this could contribute to the increased risk of infection. Another independent risk factor is a higher American Society of Anesthesiologists score (ASA score) (Ridgeway et al. 2005, Pulido et al. 2008). In our register, ASA score was registered from 2005; thus, we only have data from the last 3 years of the study period (The Norwegian Arthroplasty Register 2008). During this short period, however, we found an increase in patients with higher ASA scores. There was an increase in mean age from the first to the last time period for the uncemented THAs, but this was adjusted for in the analyses. However, age was not found to be a statistically significant risk factor concerning risk of revision due to infection.

Parvizi et al. (2008a) reported on “the changing organism profile in periprosthetic infection”, which is another risk factor not recorded in the NAR. The microbes causing periprosthetic infections could have become more virulent or resistant (Styers et al. 2006, Anderson et al. 2007). More extensive use of antibiotic prophylaxis systemically and in bone cement may have resulted in selection of more virulent or resistant microbes (Santos Sanches et al. 2000).

The clinical presentation of aseptic loosening and low-grade periprosthetic infection can be similar (Ince et al. 2004). After revision surgery the diagnosis, reported immediately after surgery, will be based on preoperative blood and bacterial samples and peroperative evaluation by the surgeon. Unexpectedly positive peroperative bacterial cultures will be recognized postoperatively and are not reported to NAR. An incorrect reported diagnosis will therefore not be corrected in the register. Improved diagnostics and knowledge about the ability of microbes to cause infection would only affect our results if, with time, preoperative bacterial detection improved or changed surgeons’ evaluation of the clinical diagnosis.

There have been improvements in procedures for diagnosis of periprosthetic infection, and more standardized techniques of sampling, culture, and analysis lead to less samples being false negative (Dempsey et al. 2007, Moojen et al. 2007, Neut et al. 2007). Also, bacteria such as Staphylococcus epidermidis have emerged as important agents of implant infection (Neu 1994, Raad et al. 1998, von Eiff et al. 2006, Anderson et al. 2007). Earlier in the period studied, these species were considered to be incapable of causing infections. This may have led to deep infection being suspected, and therefore reported, more frequently in recent years. The magnitude of this shift remains unclear, but with 4,437 revisions due to aseptic loosening and only 614 revisions due to infection, even small improvements in diagnostics and in our understanding of low-grade infections may have had an influence on the results. However, we found no change in percentage revision due to aseptic loosening between the last 2 time periods, whereas it was between these two time periods that we found the greatest increase in percentage revision due to deep infection (Figure 3).

We do not have information on what time the systemically administered antibiotics were given prior to surgery, or if there were changes in this routine over time. This has been shown to be of importance concerning the protective ability of antibiotic prophylaxis (van Kasteren et al. 2007). These factors may have influenced our results.

Because of the large numbers and the long period of observation, registry studies on deep infection can be a useful source of information regarding incidences and trends. The NAR has good-quality, detailed information about patients, primary surgery, and prophylactic measures, gathered uniformly over a long period of time. Our data are prospective, with 95–97% completeness for primary THA (Havelin 1995, Espehaug et al. 2006). We therefore have an excellent basis for a trend study on a relatively rare complication like periprosthetic infection. However, with 97,344 THAs available for analysis, there were only 614 revisions due to infection available for analysis. This restricts division into subgroups, and when this is done, marginal effects are difficult to assess.

Registry results are influenced by confounding factors. Changes in reporting, revision policy, diagnostics, surgeon awareness and surgery, selection of patients, and the virulence of microbiotic agents will also influence the results. These factors can only be partially elucidated. Completeness studies on the NAR have shown that there is 10–20% under-reporting of Girdlestone procedures, which is a common procedure in revision surgery for deep infection (Arthursson et al. 2005, Espehaug et al. 2006). These procedures will, however, be registered if a second stage in the revision is performed and reported. Under-reporting will only affect our findings if the degree to which it happens changes over the period studied. Awareness of the importance of thorough reporting probably improved the reporting of infection over the study period, but a time trend evaluation of this was not done.

We found an increase in the risk of revision due to infection during the first postoperative year for the 2002–2007 group. This shows that the infections were revised earlier after index surgery in recent years. This can either be explained by a change in revision policy, a change in surgeons’ awareness, or more acute infections. Current recommendations for early surgical site infection involve early soft tissue debridement and exchange of prosthesis parts (Zimmerli and Ochsner 2003). In our material, we found a shift from use of monoblock prostheses to more frequent use of modular prostheses. Early revision due to infection in the case of modular prostheses will therefore involve the exchange of a femoral head, an acetabular liner, or both, and the procedure should therefore be reported to the registry. Early revisions for infection in the case of monoblock prostheses will not, however, be reported if a successful soft tissue debridement combined with antibiotic treatment heals the infection and the prosthesis is retained. We adjusted for monoblock or modular prosthesis in our Cox analysis, to adjust for changes in reporting of deep infection due to these changes in the use of implants. In addition, because of the possible “under-reporting” of deep infection in the monoblock group, we also performed separate analyses on Charnley monoblock prostheses and found an increase in risk of infection in this group as well.

Improvements in the design of prostheses and surgical technique have reduced the incidence of aseptic loosening in recent years (Herberts and Malchau 2000, Morscher 2003). This could affect surgeons’ awareness of low-grade infection when deciding on the clinical diagnosis to report after surgery.

The problem of confounding factors and time-dependent risk factors in our registry study is the reason why we must interpret the evaluation of the risk factors and prophylactic measures in Table 3 with caution. The evaluation was made to illustrate the effect of these factors in this study, and the study was not set up to assess each covariate independently.

Due to the small numbers of infections, large numbers of primary THAs are needed to study different aspects of periprosthetic infections. There is a need for improved monitoring of time trends and evaluation of prophylactic measures concerning deep infection. For this purpose, surveillance programs such as the National Nosocomial Infections Surveillance (NNIS) System Reports (USA) and the European surveillance HELICS-SSI database could be of value, as could the increasing number of national arthroplasty registries and improved collaboration between these. Concentration on and improvement of prophylaxis, diagnostics, and treatment of these infections will be of great importance to limit any increase in this serious complication.
